# A new species of Dactylolabis subgenus Dactylolabis Osten Sacken, 1860 from China (Diptera, Limoniidae)

**DOI:** 10.3897/zookeys.1047.62033

**Published:** 2021-06-28

**Authors:** Shang Gao, Bing Zhang, Ding Yang

**Affiliations:** 1 Department of Entomology, College of Plant Protection, China Agricultural University, Beijing 100193, China China Agricultural University Beijing China

**Keywords:** Biodiversity, crane flies, Dactylolabinae, key, taxonomy

## Abstract

Only two species of Dactylolabis subgenus Dactylolabis Osten Sacken, 1860 were previously known from China. Here, a new species, Dactylolabis (Dactylolabis) wudangensis**sp. nov.**, is reported from China. Dactylolabis (D.) gracilistylus Alexander, 1926 is re-described and illustrated. A key to males of species of the subgenus Dactylolabis from China is presented.

## Introduction

Dactylolabis subgenus Dactylolabis Osten Sacken, 1860 (Diptera, Limoniidae) is a large subgenus in the subfamily Dactylolabinae. It is distributed worldwide with 50 known species, of which 32 taxa are from the Palaearctic Region, including 16 from Europe, and 18 from the Nearctic Region (Oosterbroek 2021). The subgenus is characterized by the following features: antennae 16-segmented; vein *MA* missing; crossvein *m-cu* near base of cell *dm*; outer gonostylus fleshy with many setae; cerci of ovipositor with wide apex (Osten Sacken 1860; Savchenko 1978; Alexander and Byers 1981; Starý 1992; Podenas et al. 2006; Ribeiro 2008).

## Materials and methods

The specimens were studied and illustrated with a ZEISS Stemi 2000-c stereomicroscope. Details of the coloration were checked in specimens immersed in 75% ethyl alcohol (C_2_H_5_OH). Genitalic preparations of males were made by macerating the apical portion of the abdomen in cold 10% NaOH for 12–15 hours. After examination, the genitalia were transferred to fresh glycerine (C_3_H_8_O_3_) and stored in a microvial pinned below the specimen. Type specimens of the new species are deposited in the Entomological Museum of China Agricultural University, Beijing, Chin (**CAU**). The holotype of D. (D.) mokanica Alexander, 1940 was borrowed from the Institute of Zoology, China Academy of Sciences, Beijing, China (**IZCAS**).

The morphological terminology mainly follows McAlpine (1981), Alexander and Byers (1981), and Savchenko (1978). The terminology applied to the wing veins follows the interpretations of Savchenko (1978) and de Jong (2017). Terminology of the male hypopygium follows Savchenko (1978) and Alexander and Byers (1981). The following abbreviations are used: og = outer gonostylus, ig = inner gonostylus, aed = aedeagus, gx = gonocoxite, 9t = ninth tergite, 9s = ninth sternite.

## Taxonomy

### A key to adult males of the subgenus Dactylolabis from China

**Table:** 

1	Wing yellowish hyaline throughout, except pterostigma (Figs 1, 3); vein *R_4_* relatively straight at tip (Figs 1, 3; Alexander 1926: pl. 1, fig. 8)	**Dactylolabis (Dactylolabis) gracilistylus Alexander, 1926**
–	Wing yellowish hyaline with brownish markings, except pterostigma (Figs 10, 11, 17); vein *R_4_* relatively curved at tip (Figs 10, 11, 17; Alexander 1940: p. 22, fig. 12)	**2**
2	Cell *r_1_* not broad at pterostigma; crossvein *sc-r* shorter than vein *R_1_*; crossvein *m-cu* near 1/3 of cell dm (Fig. 10; Alexander 1940: p. 22, fig. 12) ; tips of veins *A_1_* and *Cu P* with brownish markings (Fig. 10)	**Dactylolabis (Dactylolabis) mokanica Alexander, 1940**
–	Cell *r_1_* rather broad at pterostigma; crossvein *sc-r* longer than vein *R_1_*; crossvein *m-cu* near 1/5 of cell *dm*; tips of veins *A_1_* and *Cu P* without brownish markings (Figs 11, 17)	**Dactylolabis (Dactylolabis) wudangensis sp. nov.**

#### Dactylolabis (Dactylolabis) gracilistylus

Taxon classificationAnimaliaDipteraTipulidae

Alexander, 1926

89C74502-1E0C-578B-BD07-F038CC40A959

Figs 1–3 , 4–7

Dactylolabis gracilistylus Alexander, 1926: 372. Type locality: China: Zhejiang.

##### Diagnosis.

Wing yellowish hyaline, pterostigma brownish. Vein *R_3_* as long as vein *R_2+3_*. Veins *R_4_* and *R_5_* relatively straight. Vein *M_1_* as long as vein *M_1+2_*. Crossvein *m-cu* located before or near base of cell *dm.* Posterior margin of 9t with an M-shaped process and a shallow V-shaped notch at middle. Inner gonostylus slender, curved; gonocoxite very elongate and slender, more than twice as long as outer gonostylus. Aedeagus very big, with a shallow V-shaped notch at posterior margin.

##### Redescription.

**Male** (*n* = 3). Body length 8.2–8.5 mm, wing length 8.4–8.8 mm, antenna length 1.6–1.7 mm.

**Figures 1–3.**
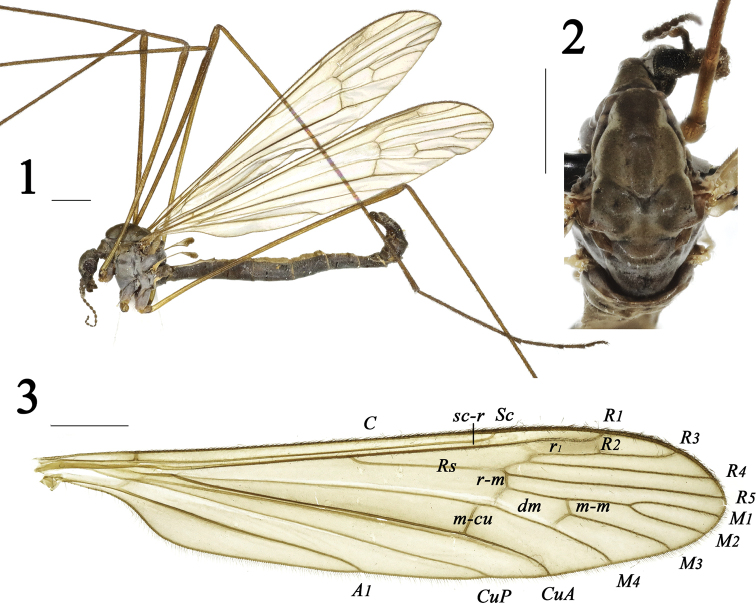
Dactylolabis (Dactylolabis) gracilistylus Alexander, 1926, male **1** habitus, lateral view **2** head and thorax, dorsal view **3** right wing. Scale bars: 1 mm.

***Head*** (Figs 1, 2) dark brown with pale gray pollen. Vertex with long setae. Rostrum and palpus brown. Antenna brown.

***Thorax*** (Figs 1, 2) mostly dark brown with gray pollen. Pronotum rather long; mesonotum brownish, prescutum dark brown with pale gray pollen. Thoracic pleuron mostly dark brown with dense gray pollen. Legs: coxae brown with gray pollen; trochanters brownish-yellow; femora more yellow at base, brownish-yellow at tip; tibiae brownish-yellow; tarsi brown. Wing (Figs 1, 3) yellowish hyaline, pterostigma more brownish; veins brownish. Venation: *Rs* long; *R_2_* relatively oblique; *R_3_* as long as *R_2+3_*; *R_4_* and *R_5_* relatively straight; *M_1_* as long as *M_1+2_*; *m-cu* located before or near base of cell *dm.* Halter (Fig. 1) length approximately 1.3 mm, halter stem yellowish; halter brownish.

***Abdomen*** (Fig. 1) elongated, tergites brownish-yellow, sternites dark brown.

**Figures 4–7:**
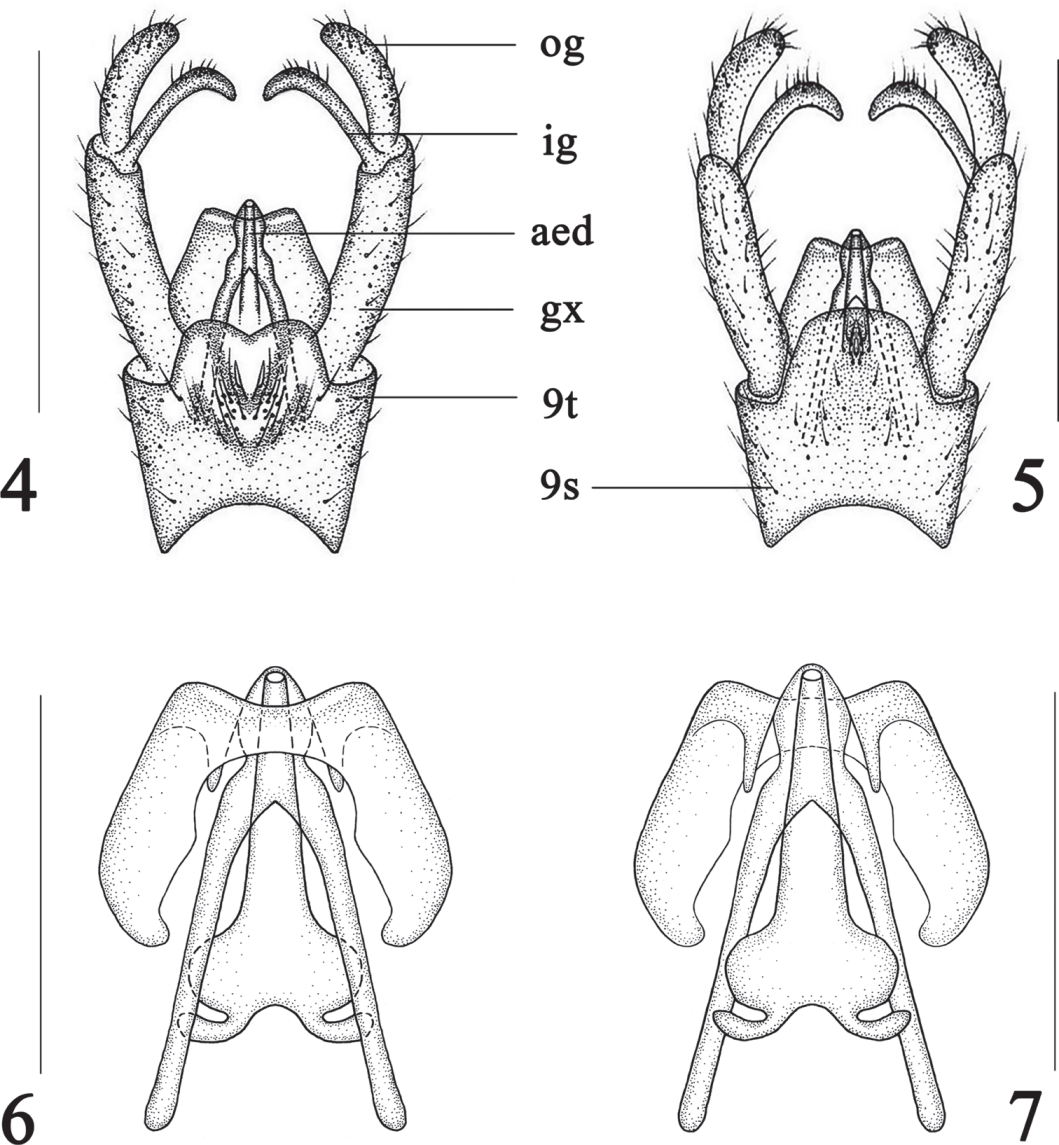
. Dactylolabis (Dactylolabis) gracilistylus Alexander, 1926, male **4** hypopygium, dorsal view **5** hypopygium, ventral view **6** aedeagal complex, dorsal view **7** aedeagal complex, ventral view. Scale bars: 1 mm (**4, 5**); 0.5 mm (**6, 7**).

***Hypopygium*** (Figs 1, 4–7) dark brown with brownish setae. Surface of 9t with plenty of long setae, posterior margin with an M-shaped process, medially with a shallow V-shaped notch; posterior margin of 9s with plenty of long setae; outer gonostylus cylindrical; inner gonostylus slender, curved; gonocoxite very elongate and slender, more than twice as long as outer gonostylus; aedeagus hyaline, very big, posterior margin with a shallow V-shaped notch.

**Female.** Similar to male (Alexander 1926: 372).

##### Material examined.

1 male (CAU), China: Zhejiang, Yuyao, Siming Mountain, 1980.IV.27, Jikun Yang. 1 male (CAU), China: Zhejiang, Qingyuan, Baishanzu, 1984.IV.19, Hong Wu. 1 male (CAU), China: Zhejiang, Deqing, Mogan Mountain, 1991.IV.20.

##### Distribution.

China (Zhejiang).

#### Dactylolabis (Dactylolabis) mokanica

Taxon classificationAnimaliaDipteraTipulidae

Alexander, 1940

95F1CDA7-A724-54FB-9CE8-DA7C2AB806CC

Figs 8–10

Dactylolabis mokanica Alexander, 1940: 22. Type locality: China: Zhejiang: Mogan Mountain.

##### Diagnosis.

Tips of veins *A_1_* and *Cup* with brownish markings. Vein *R_3_* as long as vein *R_2+3_*. Vein *R_4_* relatively curved at tip. Vein *M_1_* about twice as long as vein *M_1+2_*. Crossvein *m-cu* located at basal 1/3 of cell *dm*.

##### Distribution.

China (Zhejiang).

##### Material examined.

***Holotype***, male, China: “Chekiang: Mokan Shan” (= Zhejiang: Mogan Mountain), April 30, 1936, Institute of Zoology, China Academy of Sciences, accession no. IOZ(E) 201063 (IZCAS).

**Figures 8–10.**
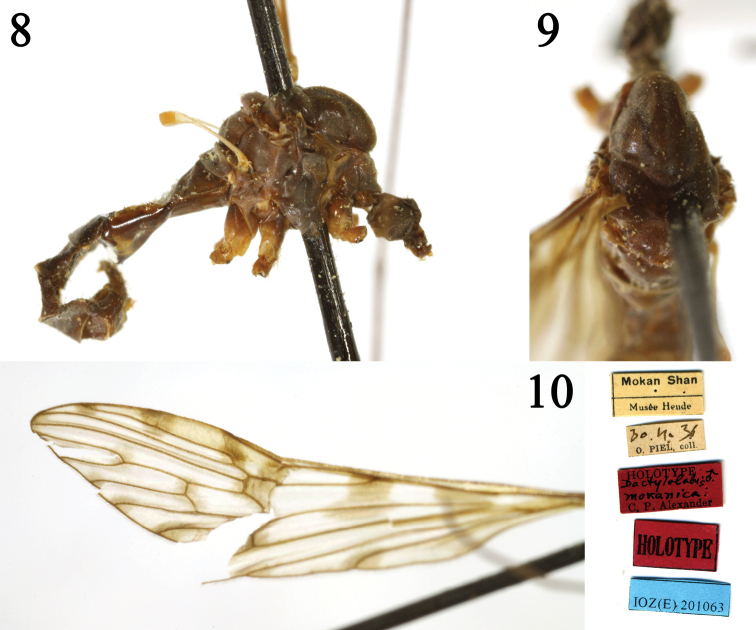
Dactylolabis (Dactylolabis) mokanica Alexander, 1940, male **8** habitus, lateral view **9** head and thorax, dorsal view **10** left wing.

#### Dactylolabis (Dactylolabis) wudangensis sp. nov.

Taxon classificationAnimaliaDipteraTipulidae

DD433089-83AA-560C-8E17-530A8523E3FC

http://zoobank.org/8882A009-75B2-4B7D-B5F6-B0A935B1CA4C

Figs 11–17 , 18–21

##### Diagnosis.

Cell *r_1_* relatively broad at pterostigma. Vein *R_3_* shorter than vein *R_2+3_*. Vein *R_4_* relatively curved at tip. Vein *M_1_* about twice as long as vein *M_1+2_*. Crossvein *m-cu* located at 1/5 of cell *dm.* Posterior margin of 9t with an M-shaped process and a deep V-shaped notch at middle. Inner gonostylus stubbier than outer gonostylus, curved. Gonocoxite rather short, as long as outer gonostylus. Aedeagus very big, with an elongated tip at posterior margin.

##### Description.

**Male** (*n* = 3). Body length 7.2–10.1 mm, wing length 14.2–18.8 mm, antenna length 2.2–2.4 mm.

**Figures 11–17.**
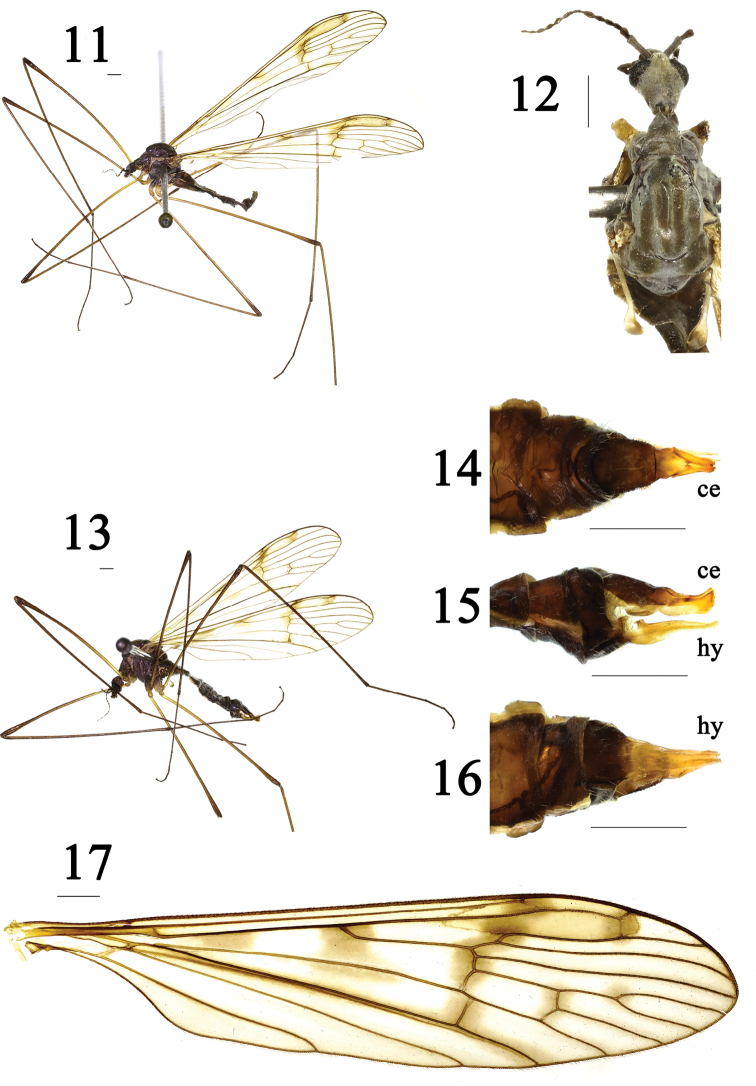
Dactylolabis (Dactylolabis) wudangensis sp. nov. **11** male habitus, lateral view **12** male head and thorax, dorsal view **13** female habitus, lateral view **14** ovipositor, dorsal view **15** ovipositor, lateral view **16** ovipositor, ventral view **17** male right wing. Scale bars: 1 mm.

***Head*** (Figs 11, 12) dark brown with gray pollen. Rostrum and palpus brown. Antenna brown.

***Thorax*** (Figs 11, 12) mostly dark brown with gray pollen. Pronotum rather long; mesonotum brownish, prescutum brown with four dark brown stripes. Thoracic pleuron mostly dark brown with pale gray pollen. Legs: base of coxae brown, tip of coxae and trochanters brownish-yellow; femora more yellow at base, brown at tip; tibiae and tarsi brown. Wing (Figs 11, 17) yellowish hyaline, pterostigma more brownish, and with brownish markings near base of wing, origin of *Rs*, around crossvein *sc-r* and vein *R_2_*, base of vein *R_4_*, crossveins *r-m* and *m-m*, crossvein *m-cu*, and vein *CuA*; veins brown. Venation: cell *r_1_* relatively broad at pterostigma; *Rs* long; *R_2_* relatively straight; *R_3_* shorter than *R_2+3_*; *R_4_* relatively curved at tip; *R_5_* relatively straight; *M_1_* about twice as long as *M_1+2_*; *m-cu* located at 1/5 of cell *dm.* Halter (Figs 11, 12) approximately 2.2 mm long, stem yellowish, rest gray.

***Abdomen*** (Fig. 11) mostly dark brown with brownish-yellow setae.

**Figures 18–21.**
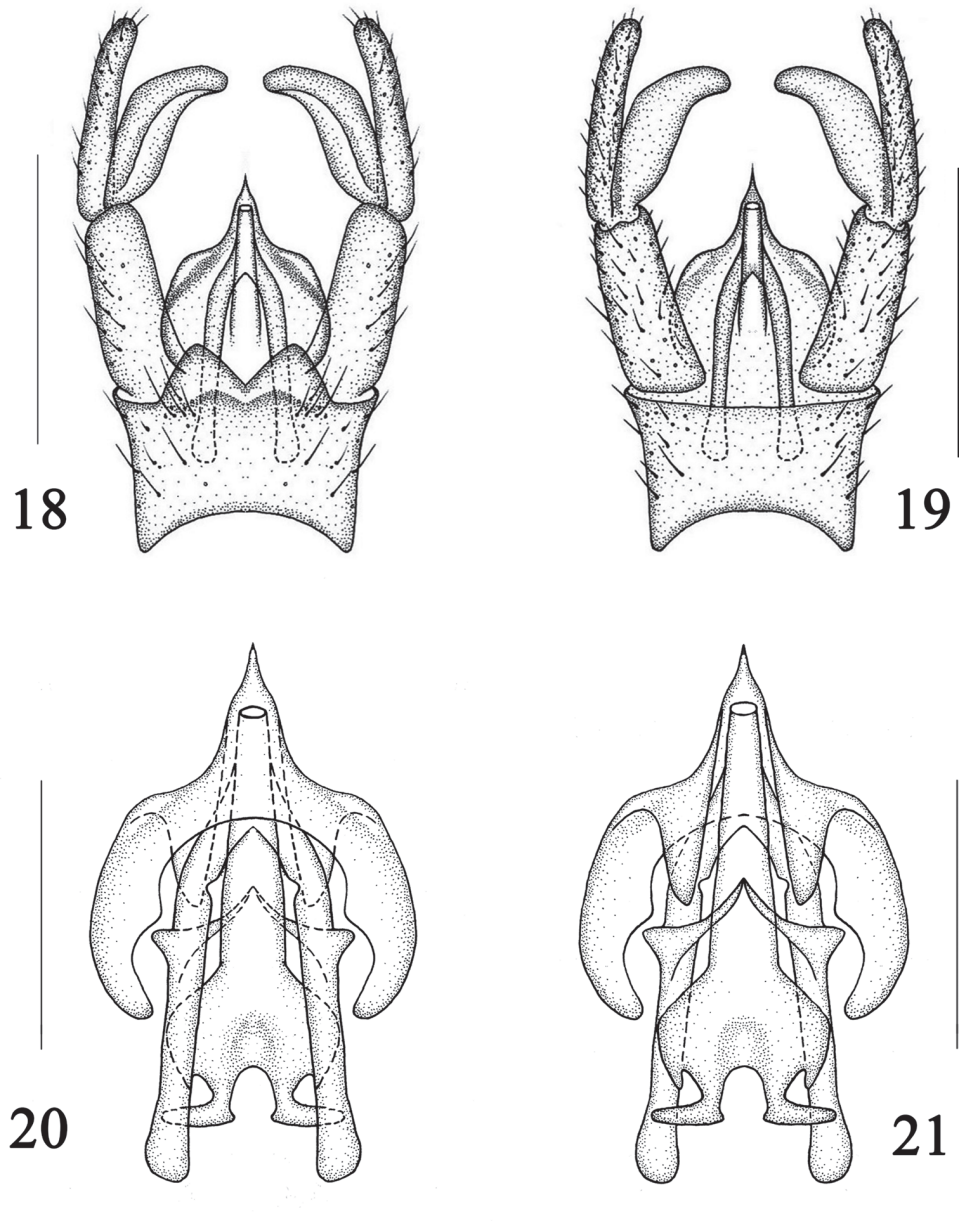
Dactylolabis (Dactylolabis) wudangensis sp. nov., male **18** hypopygium, dorsal view **19** hypopygium, ventral view **20** aedeagal complex, dorsal view **21** aedeagal complex, ventral view. Scale bars: 1 mm (**18, 19**); 0.5 mm (**20, 21**).

***Hypopygium*** (Figs 11, 18–21) brown with brownish setae. Surface of 9t with numerous long setae, posterior margin with an M-shaped process, with a deep V-shaped notch at middle; outer gonostylus cylindrical; inner gonostylus stubby, curved; gonocoxite rather short, as long as outer gonostylus; aedeagus hyaline, very big, with an elongated tip at posterior margin.

**Female** (*n* = 1). Similar to male. Body length 8.6 mm, wing length 13.5 mm, antenna length 2.3 mm.

***Ovipositor*** (Figs 13–16) brown with yellow setae. Cercus reddish-brown, broadened at base. Hypogynial valve yellow, narrowed toward tip, longer than cercus.

##### Type material.

***Holotype***: male (CAU), China: Hubei, Danjiangkou, Wudang Mountain, 1600 m, 1984.VI.31, Jikun Yang. ***Paratypes***: 2 males, 1 female (CAU), China: Hubei, Danjiangkou, Wudang Mountain, 1600 m, 1984.VI.31, Jikun Yang.

##### Distribution.

China (Hubei).

##### Etymology.

The species is named after the type locality, Wudang Mountain.

##### Remarks.

The new species is somewhat similar to D. (D.) mokanica Alexander, 1940 from China (Zhejiang), but can be separated from the latter by crossvein *sc-r* slightly longer than crossvein *R_1_*, *m-cu* located at 1/5 of cell *dm*, and tips of veins *A_1_* and *CuA* without brownish markings (Figs 11, 17). In D. (D.) mokanica, crossvein *sc-r* is shorter than vein *R_1_*, crossvein *m-cu* is located at 1/3 of cell *dm*, and the tips of veins *A_1_* and *CuA* have brownish markings (Fig. 10; Alexander 1940: p. 22, fig. 12). The new species is somewhat similar to D. (D.) dilatata (Loew, 1856) from the West Palearctic and D. (D.) subdilatata Starý, 1969 from Czechia in having similar wing markings and venation, but can be separated from the latter two species by posterior margin of 9t with an M-shaped process and cercus shorter than hypogynial valve (Figs 13–16, 18, 19). In D. (D.) dilatata and D. (D.) subdilatata, the posterior margin of 9t lacks an M-shaped process and the cercus is longer than the hypogynial valve (Stary 1969: p. 125, figs 1, 4, 5, 8). The new species is somewhat similar to D. (D.) dilatatoides Savchenko, 1978 from Kazakhstan in having similar wing markings, but can be separated from the latter by vein *R_2+3+4_* as long as vein *R_2_*, and posterior margin of 9t with an M-shaped process (Figs 11, 17–19). In D. (D.) dilatatoides, vein *R_2+3+4_* is almost absent and the posterior margin of 9t has a deep V-shaped notch (Savchenko 1978: p. 1176, fig. 1; p. 1177, fig. 3). The new species is somewhat similar to D. (D.) laticellula Savchenko, 1978 from Russia in having similar wing venation, but can be separated from the latter by wing with brownish markings and posterior margin of 9t with an M-shaped process (Figs 11, 17–19). In D. (D.) laticellula, the wing has no markings and the posterior margin of 9t lacks an M-shaped process (Savchenko 1978: p. 1176, fig. 2; p. 1177, fig. 4).

## Supplementary Material

XML Treatment for Dactylolabis (Dactylolabis) gracilistylus

XML Treatment for Dactylolabis (Dactylolabis) mokanica

XML Treatment for Dactylolabis (Dactylolabis) wudangensis
